# Effects of Sacubitril-Valsartan in Heart Failure With Preserved Ejection Fraction in Patients Undergoing Peritoneal Dialysis

**DOI:** 10.3389/fmed.2021.657067

**Published:** 2021-06-21

**Authors:** Sha Fu, Zhenjian Xu, Baojuan Lin, Junzhe Chen, Qiuyan Huang, Yanchun Xu, Anping Xu, Yangxin Chen, Ying Tang

**Affiliations:** ^1^Department of Nephrology, Guangdong Provincial Key Laboratory of Malignant Tumor Epigenetics and Gene Regulation, Sun Yat-Sen Memorial Hospital, Sun Yat-Sen University, Guangzhou, China; ^2^Departments of Medicine & Therapeutics, Li Ka Shing Institute of Health Sciences, and Lui Che Woo Institute of Innovative Medicine, The Chinese University of Hong Kong, Hong Kong, China; ^3^Department of Cardiology, Guangdong Provincial Key Laboratory of Malignant Tumor Epigenetics and Gene Regulation, Sun Yat-Sen Memorial Hospital, Sun Yat-Sen University, Guangzhou, China; ^4^Department of Nephrology, The Third Affiliated Hospital, Southern Medical University, Guangzhou, China

**Keywords:** sacubitril-valsartan, heart failure, preserved ejection fraction, peritoneal dialysis, NT-ProBNP

## Abstract

**Aims:** The effect of the angiotensin receptor–neprilysin inhibitor (ARNI) sacubitril-valsartan in patients with heart failure with preserved ejection fraction (HFpEF) remains unclear, and data on ARNI treatment in peritoneal dialysis (PD) patients are lacking. The present study was designed to assess the efficacy and safety of sacubitril-valsartan in patients with HFpEF undergoing peritoneal dialysis.

**Methods and Results:** End-stage kidney disease (ESKD) patients undergoing PD for 3 months with New York Heart Association (NYHA) class II–IV heart failure, ejection fraction of 50% or higher, and elevated levels of N-terminal pro–B-type natriuretic peptide (NT-proBNP) were assigned to receive sacubitril-valsartan. Patients were followed up regularly after medication treatment. The alterations in clinical and biochemical parameters before and after taking sacubitril-valsartan (generally 50–100 mg b.i.d) were investigated, and safety was also assessed. Twenty-one patients were recruited in this study. Compared with baseline levels, NT-proBNP levels [9769.0 (3093.5–21941.0) vs. 3034.0 (1493.2–6503.0), *P* = 0.002], and heart rate [80.0 (74.5–90.5) vs. 75.0 (70.3–87.0), *P* = 0.031] were markedly decreased after treatment with sacubitril-valsartan. Signs and symptoms of heart failure (21/21 vs. 15/21, *P* = 0.021) were obviously alleviated, NYHA classification and E/e' ratio showed a notable trend of improvement after 3–12 months of follow-up. None of the patients showed adverse drug reactions.

**Conclusions:** The present data suggested that sacubitril-valsartan treatment in patients with HFpEF undergoing PD was effective and safe.

## Introduction

Heart failure (HF) with preserved ejection fraction (HFpEF), also termed diastolic HF, is commonly defined as ejection fraction (EF) > 50% according to the criteria defined by the European Society of Cardiology (1); HFpEF accounts for approximately half of the cases of HF and is associated with substantial morbidity and mortality (2–4). Cheng et al. (5) showed that patients hospitalized with HFpEF experience more readmission after discharge, demonstrated as 20% readmittance within 30 days and >50% within 1 year. Although HFpEF has a poor prognosis, there are no effective medications to treat HFpEF except for diuretics, which is a stark difference from HF with reduced EF (HFrEF) (6). HFpEF is a heterogeneous clinical syndrome that could be caused by varied aetiological factors, including ageing, obesity, coronary heart disease, diabetes, hypertension, and renal impairment (7). The pathophysiology of HFpEF remains incompletely understood, and cardiomyocytes, extracellular matrix, inflammation, and peripheral vasculature may contribute to the aetiology of diastolic HF (7, 8). In particular, patients with chronic kidney disease (CKD) are at increased risk of HF and are associated with worse outcomes (9, 10). Researchers (11) have found that CKD was more common in HFpEF than in HF with mid-range EF (HFmrEF) and HFrEF. Wang et al. (12) also showed that HFpEF is common in peritoneal dialysis (PD) patients (accounting for ~55% of all HF), and is associated with an increased risk of mortality and poor cardiovascular outcomes in these patients compared with PD patients without HF.

Recently, PARADIGM-HF Clinical Trials demonstrated that the angiotensin receptor–neprilysin inhibitor LCZ696 was superior to enalapril in reducing the risks of death and hospitalization for patients with HFrEF (13). Notably, subgroup analysis demonstrated that sacubitril-valsartan also led to a slower decrease in eGFR in CKD patients with HFrEF (14). However, the effect of sacubitril-valsartan on HFpEF remains controversial. The PARAMOUNT-HF trial demonstrated that ARNI resulted in a lower level of N-terminal pro–B-type natriuretic peptide (NT-proBNP), a larger reduction in left atrial size, and greater improvement in the New York Heart Association (NYHA) functional class than valsartan (15). In contrast, recent data from the PARAGON-HF trial did not demonstrate a positive protective role of sacubitril/valsartan on hospitalizations for HF and death from cardiovascular causes among patients with an EF of 45% or higher (16). At present, data on patients with severe renal insufficiency treated with sacubitril/valsartan are lacking, since patients with severe renal insufficiency with a glomerular filtration rate (GFR) below 30 ml/min/1.73 m^2^ of body surface area are usually excluded from trials. Thus, we undertook this study to investigate the effects of sacubitril/valsartan on patients with HFpEF undergoing PD.

## Methods

### Study Design and Patients

This was a retrospective, self-controlled, observational study. Eligible patients in this study were 18 years or older with chronic HFpEF who underwent PD, and were referred to the Department of Nephrology in the PD Center of Sun Yat-sen Memorial Hospital between January 2018 and December 2019. The patients with end-stage kidney disease (ESKD) received percutaneous PD catheter insertion in our hospital and had been undergoing PD for more than 3 months. The PD modality was continuous ambulatory PD (CAPD) using glucose-containing dialysis fluid, which was exchanged four or five times daily. Inclusion criteria must simultaneously meet with following conditions: ESKD patients with residual renal function and undergoing CAPD > 3 months, experienced one or more episode of HF that required hospitalization. The exclusion criteria were as follows: acute coronary syndrome and pulmonary-associated disease including asthma attack, pulmonary embolism, or chronic obstructive pulmonary disease, inadequate PD, including irregular dialysis, overt hypervolemia, symptomatic hypotension or systolic blood pressure <100 mmHg at screening, and poor compliance with follow-up during the period of sacubitril-valsartan treatment. Patients with signs and symptoms of HF, NYHA class II–IV, an EF of 50% or higher within the previous 6 months, and elevated levels of NT-proBNP were prescribed sacubitril-valsartan. Sacubitril-valsartan was administered after consultation with the cardiologist and after informed oral consent of the patients together with previously prescribed medication for complications related to ESKD, including diabetes, hypertension, anaemia, and secondary hyperparathyroidism. Furthermore, ACE inhibitors were required to be discontinued 36 h before prescribing sacubitril/valsartan, and ARBs were discontinued except for three patients who received a low dose of valsartan simultaneously. No adverse reactions such as hyperpotassium and hypotension occurred in the above three patients. In this study, patients prior prescribed with aldosterone-antagonists continued to take as usual. Sacubitril-valsartan was progressively titrated, starting from a low dose to a tolerable maximum (generally 50–100 mg b.i.d), and no patients discontinued the drug during the follow-up. All patients were required to undergo serum potassium and creatinine tests once a week until stabilization, and were followed up for recurrent hospitalizations for HF and death from cardiovascular causes. The study protocol was submitted to our hospital's ethics committee and approved.

### Data Collection

At baseline prior to drug administration, demographic and clinical parameters including age, sex, body mass index (BMI), duration of PD, Kt/V (weekly fractional clearance index for urea), primary renal disease, medical history, laboratory data, and medication use were obtained from the medical records and local laboratory analysis. Clinical parameters including blood pressure, heart rate, signs, and symptoms (defined as dyspnoea on effort, paroxysmal nocturnal dyspnoea, orthopnoea, oedema, rales, and third heart sound), and 24 h urine volume were collected.

Cardiac structure and function were assessed by two-dimensional echocardiography and NYHA functional class. There were two observers who carried out the echocardiographic measurements. The echo reports were cross-checked by two independent investigators. Parameters included left ventricular EF, E/e' ratio, TR (tricuspid regurgitation peak velocity), aortic dimension (AOR), ascending aorta (AAO), left atrium (LA), left ventricular diastolic diameter (LVDd), interventricular septum diastolic thickness (IVSd), and right ventricular diastolic diameter (RVDd). Additionally, cardiac biomarkers, including NT-proBNP, creatine kinase MB (CK-MB), cardiac troponin I and cardiac troponin T, were analysed. Chest radiography indexes were also obtained, such as cardiomegaly, interstitial or alveolar oedema, pleural effusion, vascular prominent hilum, and haziness of pulmonary vessels.

Adverse effects included hypotension (defined as a systolic blood pressure <100 mmHg), elevation of serum creatinine or decreased estimated GFR, hyperkalaemia, and angio-oedema.

The clinical parameters were collected in the same manner as above after sacubitril-valsartan prescription for at least 3 months.

### Statistical Analysis

All statistical analyses were performed using Statistical Package for the Social Sciences (SPSS) version 20.0 for Windows (SPSS Inc., Chicago, IL, United States). Descriptive results of continuous variables are presented as medians and interquartile ranges (IQRs), and categorical variables are reported as percentages and numbers. For normally distributed quantitative data, paired samples *t*-test was employed to compare self-matching data, and for non-parametric data, the Wilcoxon matched-pair signed-rank (two samples) test was applied. Qualitative data were analysed using the chi-square text (Fisher's exact test). All tests were two-tailed, and a *P* < 0.05 was considered statistically significant.

## Results

### Baseline Characteristics of the Study Subjects

From January 2018 to December 2019, 21 PD patients were recruited to participate in this study, and their baseline demographic, clinical, and laboratory characteristics are shown in Table 1. The mean age was 55.0 (38.0–61.0) years, male/female proportion was 14/7, mean BMI was 23.9 (21.0–26.2) kg/m^2^, and the mean duration of PD was 16 (6–23) months. The underlying kidney diseases were chronic glomerulonephritis (38.1%), diabetic kidney disease (23.8%), hypertensive nephropathy (14.3%), obstructive nephropathy (9.5%), and others (14.3%).

**Table 1:** Baseline characteristics of PD patients initially presenting before sacubitril-valsartan treatment.

**Variables**	**All patients (*n* = 21)**
**DEMOGRAPHICS**	
Age, years	55.0 (38.0–61.0)
Gender, male/female	14/7
BMI, kg/m^2^	23.9 (21.0–26.2)
Duration of PD, months	16 (6–23)
Weekly Kt/V	1.8 (1.7–2.0)
**CAUSES OF ESKD**	
Chronic glomerulonephritis	8 (38.1)
Diabetic kidney disease	5 (23.8)
Hypertensive nephropathy	3 (14.3)
Obstructive nephropathy	2 (9.5)
Others	3 (14.3)
**MEDICAL HISTORY**	
Hypertension	21 (100)
Diabetes mellitus	5 (23.8)
Stroke	3 (14.3)
Previous myocardial infarction	2 (9.5)
Coronary heart disease	3 (14.3)
**LABORATORY VALUES**	
Cholesterol, mmol/L	4.47 (3.7–5.0)
Triglyceride, mmol/L	1.3 (1.1–1.7)
LDL-C, mmol/L	2.9 (2.2–3.2)
HDL-C, mmol/L	1.0 (0.8–1.2)
Apolipoprotein A, mmol/L	1.1 (0.9–1.3)
Uric acid, mmol/L	419.0 (352.0–517.5)
Albumin, g/L	29.3 (26.1–32.6)
HbA1c, %	5.1 (5.0–6.3)
**MEDICATION USE**	
Calcium channel blocker	21 (100)
ACE inhibitor or ARB	6 (28.6)
Beta-blocker	11 (52.4)
Diuretics	10 (47.6)
MARs	7 (33.3)
α-blocker	10 (47.6)

*Values are proportion or median and interquartile range (IQR). PD, peritoneal dialysis; ESKD, end-stage kidney disease; Kt/V, weekly fractional clearance index for urea; ACE inhibitor, angiotensin-converting enzyme inhibitors; ARB, angiotensin II receptor blocker*.

### Comparison of the Characteristics of PD Patients Before and After Initiating Sacubitril-Valsartan

**Figure 1:**
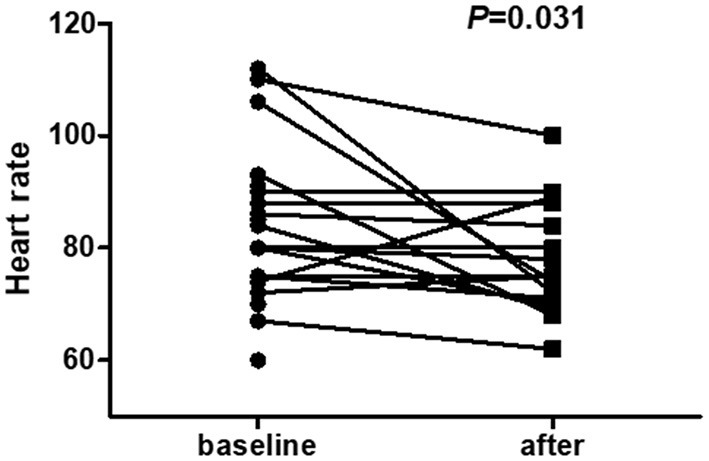
Heart rate of PD patients before and after initiating sacubitril-valsartan with an observation period of 3–12 months. Paired samples *t*-test was employed to compare self-matching data on heart rate levels. Results show that compared with baseline levels, heart rate is markedly decreased after treatment with sacubitril-valsartan [80.0 (74.5–90.5) vs. 75.0 (70.3–87.0), *P* = 0.031].

**Figure 2:**
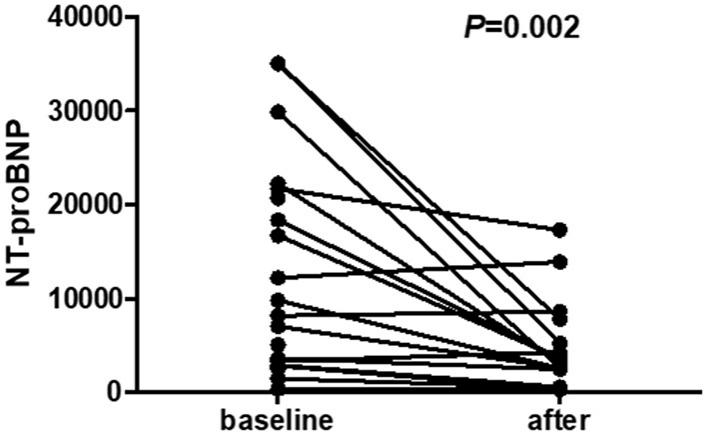
NT-proBNP levels of PD patients before and after initiating sacubitril-valsartan with an observation period of 3–12 months. Wilcoxon matched-pair signed-rank (two samples) tests were applied to compare self-matching data on NT-proBNP. Results show that compared with baseline levels, NT-proBNP levels are markedly decreased after treatment with sacubitril-valsartan [9769.0 (3093.5–21941.0) vs. 3034.0 (1493.2–6503.0), *P* = 0.002].

Twenty-one PD patients completed the self-comparison in terms of starting sacubitril-valsartan treatment. After 3–12 months of follow-up, compared with baseline levels, signs and symptoms of HF, including dyspnoea, paroxysmal nocturnal dyspnoea and orthopnoea, were obviously alleviated (21/21 vs. 15/21, *P* = 0.021), and heart rate was significantly lower than before starting sacubitril/valsartan (*P* = 0.031) (Table 2 and Figure 1). Moreover, NYHA classification showed a notable trend of improvement after 3–12 months of follow-up, although it was not statistically significant, possibly because of the small sample size (Table 2 and Supplementary Figure 2). Most importantly, NT-proBNP levels were markedly reduced after treatment with sacubitril-valsartan (*P* = 0.002) (Table 2, Figure 2, and Supplementary Figure 1). No significant differences existed, including systolic BP, diastolic BP, serum creatinine, serum potassium, phosphorus, eGFR, and echocardiography parameters, including LVEF (63 vs. 66%), E/e' (17.3 vs. 14.0), TR (257 vs. 237), AOR (21 vs. 21), LA (37 vs. 38), RVDd (20 vs. 21), IVSd (12 vs. 12), and LVDd (52 vs. 50), among patients before and after drug initiation.

**Table 2:** Comparisons of the characteristics of PD patients before and after initiating sacubitril-valsartan with observation period of 3–12 months.

**Variables**	**Before sacubitril-valsartan**	**After sacubitril-valsartan**	***P-*value**
**CLINICAL PARAMETERS**			
SBP, mmHg	144.0 (138.0–157.0)	151.0 (124.2–162.0)	0.925
DBP, mmHg	87.0 (75.5–101.5)	88.5 (76.5–102.8)	0.975
Heart rate, b.p.m	80.0 (74.5–90.5)	75.0 (70.3–87.0)	0.031
Signs and symptoms	21/21	15/21	0.021
24 h urine volume, ml	700.0 (225.0–1050.0)	700.0 (362.5–875.0)	0.061
**LABORATORY VALUES**			
Creatinine, μmoL/L	945.0 (790.0–1091.0)	945.0 (674.0–1218.0)	0.326
eGFR, ml/min/1.73 m^2^	4.6 (3.9–6.5)	4.4 (3.7–6.8)	0.552
UACR, mg/g	2190.5 (1514.1–3933.0)	1670.4 (1217.2–5019.5)	0.345
iPTH, pg/ml	438.0 (211.0–597.0)	443.0 (215.0–914.0)	0.834
Calcium, mmoL/L	2.0 (1.9–2.2)	2.1 (1.9–2.3)	0.244
Hemoglobin, g/L	81.0 (74.0–92.5)	92.0 (79.2–102.5)	0.079
Phosphorus, mmoL/L	1.8 (1.3–2.2)	1.6 (1.5–2.3)	0.802
NT-proBNP, ng/ml	9769.0 (3093.5–21941.0)	3034.0 (1493.2–6503.0)	0.002
Cardiac troponin I, μg/L	0.0 (0.0–0.4)	0.0 (0.0–0.0)	0.655
Cardiac troponin T, pg/ml	86.5 (50.2–173.9)	72.6 (34.1–136.5)	0.799
Creative kinase MB, U/L	12.0 (9.0–16.0)	12.0 (8.5–15.5)	0.929
**CARDIAC STRUCTURE AND FUNCTION**			
NYHA functional class			0.656
I	0	2	
II	7	8	
III	13	10	
IV	1	1	
LVEF, %	63.0 (54.5–68.0)	66 (49.0–71.0)	0.875
E/e' ratio	17.3 (10.3–58.0)	14.0 (10.3–36.7)	0.291
e', cm/s	5.5 (3.0–10.0)	4.0 (3.0–7.0)	0.707
TR, cm/s	257.0 (218.8–331.0)	237.0 (197.0–299.0)	0.436
LVDd, mm	52.0 (49.0–60.0)	50.0 (47.0–61.0)	0.159
AOR, mm	21.0 (20.0–22.0)	21.0 (19.8–22.3)	1.000
AAO, mm	33.0 (30.5–35.0)	33.5 (30.8–35.3)	0.173
LA, mm	37.0 (35.5–40.5)	38.0 (35.0–41.0)	0.950
RVDd, mm	20.0 (19.5–22.0)	21.0 (19.0–24.0)	0.472
IVSd, mm	12.0 (10.0–13.0)	12.0 (10.0–14.0)	0.480
**CHEST RADIOGRAPHY**			
Cardiomegaly	5/21	3/18	0.702
Interstitial or alveolar edema	0/21	0/18	–
Pleural effusion	7/21	3/18	0.290
Vascular prominent hilum	1/21	1/18	1.000
Haziness of pulmonary vessels	7/21	6/18	1.000
**ADVERSE EFFECTS**			
Hypotension	0 (0.0)	0 (0.0)	–
Hyperkalaemia	0 (0.0)	0 (0.0)	–
Angioedema	0 (0.0)	0 (0.0)	–

*Values are proportion or median and interquartile range (IQR). PD, peritoneal dialysis; SBP, systolic blood pressure; DBP, diastolic blood pressure; BUN, blood urea nitrogen; eGRF, estimated glomerular filtration rate; UACR, urinary albumin creatinine excretion rate; EF, ejection fraction; TR, Tricuspid regurgitation peak velocity; AOR, aortic dimension; AAO, ascending aorta; LA, left atrium; RVDd, right ventricular diastolic diameter; IVSd, interventricular septum diastolic thickness; RVDd, right ventricular diastolic diameter*.

### Safety of Sacubitril-Valsartan

None of the PD patients showed adverse drug reactions such as hypotension, hyperkalaemia or angio-oedema. Additionally, there was no significant change in renal function estimated by eGFR [4.6 (3.9–6.5) vs. 4.4 (3.7–6.8), *P* = 0.552] (Table 2 and Supplementary Figure 3), and serum creatinine [945.0 (790.0–1091.0) vs. 945.0 (674.0–1218.0), *P* = 0.326] (Table 2).

## Discussion

To our knowledge, this is the first report about treatment with sacubitril-valsartan in PD patients with HFpEF. Our findings demonstrated that sacubitril-valsartan significantly improved and stabilized the cardiac function of CAPD patients with HFpEF, which is supported by clinical presentation and laboratory parameters, including strengthened exercise ability, fewer signs and symptoms of HF, and decreased NT-proBNP levels and heart rate.

Substantial evidence has confirmed that HFpEF is the most common form of HF in ageing people, which accounts for a growing proportion of patients with HF and is associated with high morbidity and mortality (17, 18). Epidemiological findings have shown that HFpEF causes almost one-half of the five million cases of HF in the United States (19). Similarly, HFpEF accounts for a large proportion of hospitalized patients with HF in China according to the published data drawn from a Registry Study of 169 participating hospitals. In this study, 31,356 hospitalized patients with HF participated, including 11,034 (35.2%) patients with HFrEF, 6,825 (21.8%) patients with HFmrEF, and 13,497 (43.0%) patients with HFpEF (20). Diagnosis of HFpEF was challenging and required assessment of clinical history, physical examination, natriuretic peptide testing, echocardiography data, and invasive exercise testing (21). Recently, the Heart Failure Association (HFA) of the European Society of Cardiology (ESC) produced an updated consensus recommendation—the HFA–PEFF diagnostic algorithm including clinical assessments (HF symptoms and signs), diagnostic laboratory tests (including NT-proBNP values), and standard echocardiography (22). Notably, a combination of echocardiographic measurements of cardiac structure and function and BNP levels were recommended. Echocardiographic indicators for diagnosing HFpEF included the average septal-lateral E/e' ratio, TR (tricuspid regurgitation peak velocity), and left atrial volume index (22).

Substantial data have demonstrated CKD is an independent risk factor for cardiovascular (CV) events (23–26). The overall rate of CV disease was higher in patients with CKD as compared to those without CKD, in particular, patients with ESKD have an increased incidence of CV death ~10–20 times that of the general population. HF is more common in CKD patients (23, 27). Among hemodialysis and PD patients, the prevalence of HF is ~40% (27).

Clinical data have confirmed that HF overlapping with CKD increases the hazard ratio of hospitalization, renal replacement therapy, and death (28). Available data found that patients with ESKD are at increased risk of HF, and CKD is common in HF, especially in HFpEF compared with other forms of HF, in recently published data (11), which might result from renal dysfunction leading to elevated intracardiac filling pressures, fluid retention, and exercise intolerance (16). The available limited data disclosed that HFpEF is highly prevalent in haemodialysis (81%) (29) and PD patients (55%) (12). However, there is still no effective therapy for HFpEF in contrast with HFrEF, where angiotensin converting enzyme inhibitors (ACEIs) and angiotensin II receptor blockers (ARBs), angiotensin receptor neprilysin inhibitors, β-blockers, and mineralocorticoid receptor antagonists (MRAs) can reduce adverse outcomes associated with HFrEF (30).

The pathophysiology of HF in CKD and ESKD is very complex and includes multiple aspects associated with renal impairment: uncontrolled hypertension, left ventricular hypertrophy and fibrosis, excessive preload attributed to salt and water retention, increased afterload attributed to arterial stiffness and high output shunting through arteriovenous fistulae or grafts, neurohormonal activation, impaired iron utilization, anaemia, and bone and mineral disorders (31). Currently, neurohormones are considered to play a key role in the progression of HF in CKD patients except in preload, and left ventricular hypertrophy.

The first-in-class angiotensin receptor neprilysin inhibitor sacubitril-valsartan has been well-recognized to reduce CV and all-cause mortality, as well as the hospitalization rate in patients with HFrEF (≤40%) compared with enalapril (13). In contrast to HFrEF, whether sacubitril-valsartan plays a protective role in patients with HFpEF remains unclear. Interestingly, during the 36 weeks follow-up in 301 patients with HFpEF in the PARAMOUNT-HF trial (15), NYHA class II–III and left ventricular EF ≥45%, demonstrated a significantly greater reduction in NT-proBNP from baseline to week 12, and in left atrial size at 36 weeks with sacubitril-valsartan than with valsartan. Moreover, NYHA classification was improved at week 36. Conversely, the PARAGON-HF trial, a recent, promising double blind randomized study in 4,822 patients with HFpEF, the results showed that sacubitril-valsartan did not result in a significantly lower rate of total hospitalizations for HF and death from CV causes among patients with HF and an EF of 45% or higher (16). Nevertheless, subgroup analysis yielded a different conclusion, showing that pharmacological treatments for HFpEF seemed to reduce the risk of HF hospitalization more in women than in men (32). Noticeably, subgroup analyses of the PARADIGM-HF and PARAMOUNT-HF trials all found that sacubitril-valsartan could delay the progression of renal function deterioration in HFrEF or HFpEF patients compared to renin–angiotensin–aldosterone system (RAAS) inhibitors, although there was a modest increase in the urinary albumin/creatinine ratio (UACR) after 8 months. Furthermore, in the subgroup analysis of PARADIGM-HF, sacubitril-valsartan led to greater risk reduction in CV endpoints in patients with CKD compared to enalapril (14, 33). Notably, patients with an estimated GFR (eGFR) <30 ml/min/1.73 m^2^ were excluded in the PARAGON-HF trial as well as in the PARAMOUNT-HF trial. Recently, the UK HARP-III trial (United Kingdom Heart and Renal Protection-III) demonstrated that, in a wide range of people with proteinuric CKD, and an estimated GFR 20–60 ml/min/1.73 m^2^, sacubitril-valsartan had no extra protective effect on kidney function or albuminuria compared with irbesartan, but it could lower blood pressure and cardiac biomarker levels, including troponin I and NT-proBNP (34). Based on the above inconsistent results, the effects of sacubitril-valsartan treatment in patients with ESKD and HF are unclear, especially for PD patients with HFpEF in whom the data are null.

Recently, the effects of sacubitril-valsartan on advanced CKD patients with HFrEF were investigated in a real-world clinical setting, and the results demonstrated that the positive role of sacubitril-valsartan was supported by lower incidences of death from any cause, CV death, sudden death, and rehospitalization, including patients with advanced renal impairment (35). Thus, we evaluated, for the first time, the efficacy, safety, and tolerability of sacubitril-valsartan in patients undergoing PD with HFpEF (≥50%). In the present study, we found that the HF phenotype HFpEF seemed more common in PD patients, consistent with a previous study (11). Impressively, we observed a greater treatment effect on reducing heart rate, and cardiac marker NT-proBNP after sacubitril-valsartan use for 3 months or more. In addition, notable improvement of NYHA classification and signs and symptoms of HF, such as dyspnoea, paroxysmal nocturnal dyspnoea and orthopnoea, were found after medication use. Cardiac diastolic function showed a trend of improvement, demonstrated as a lower ratio of E/e' after treatment with ARNI and decreased by 19% compared to before treatment, although we did not find statistically significant improvements in echocardiographic indicators associated with HFpEF (E/e', TR, and LVDd), which might be related to the small sample size and short follow-up time. Available evidence has demonstrated that patients diagnosed with HFpEF are always complicated by concomitant abnormal cardiac diastolic function, which is associated with a poor prognosis due to the lack of effective therapy. In view of the safety of sacubitril/valsartan, renal function was of the greatest concern. Although patients with HFpEF in this study were in advanced renal failure, the majority of PD patients had residual renal function; thus, no obvious reduction in estimated GFR during follow-up and no severe hyperkalaemia or unstable serum creatinine was found during medication.

Some inevitable limitations also need to be considered. First, this was a small-sample, unblinded, non-prospective, single-centre study with shorter follow-up. Second, the included patients all progressed to the ESKD stage and were receiving PD, which means that volume load cannot be completely excluded, although we selected only PD patients with residual renal function demonstrated as average 24 h urine volume was 700 ml, and had already been undergoing CAPD for over 3 months without overt edema. Moreover, patients with inadequate PD and overt hypervolaemia were excluded. Furthermore, in this study, we adopted the self-control method to minimize possible confounding factors, and previous medications continued to be used. In the future, a large-sample, double-blinded, controlled study of PD patients with HFpEF should be performed to verify the effect of sacubitril/valsartan.

## Conclusions

Our study suggested the effectiveness and safety of sacubitril-valsartan in PD patients with HFpEF. This is the first study about ARNI treatment for PD patients with HFpEF, and it may bring hope for these patients due to the lack of other effective methods at present.

## Data Availability Statement

The raw data supporting the conclusions of this article will be made available by the authors, without undue reservation.

## Ethics Statement

The studies involving human participants were reviewed and approved by Ethics Committee of Sun Yat Sen Memorial Hospital. The patients/participants provided their written informed consent to participate in this study.

## Author Contributions

JC and YT acquisition, analysis, or interpretation of data. ZX and SF drafted of the manuscript. YC and YT critical revision of the manuscript. BL, JC, QH, YX, and AX statistical analysis. All authors contributed to the article and approved the submitted version.

## Conflict of Interest

The authors declare that the research was conducted in the absence of any commercial or financial relationships that could be construed as a potential conflict of interest.
