# Supplementary material for: Effects of Sacubitril-Valsartan in Heart Failure With Preserved Ejection Fraction in Patients Undergoing Peritoneal Dialysis
Source: Front Med (Lausanne). 2021 Jun 21;8:657067. doi: 10.3389/fmed.2021.657067 (PMC8255468; doi:10.3389/fmed.2021.657067)
Supplement: Supplementary file 1 [file Data_Sheet_1.pdf]

SUPPLEMENTARY MATERIAL

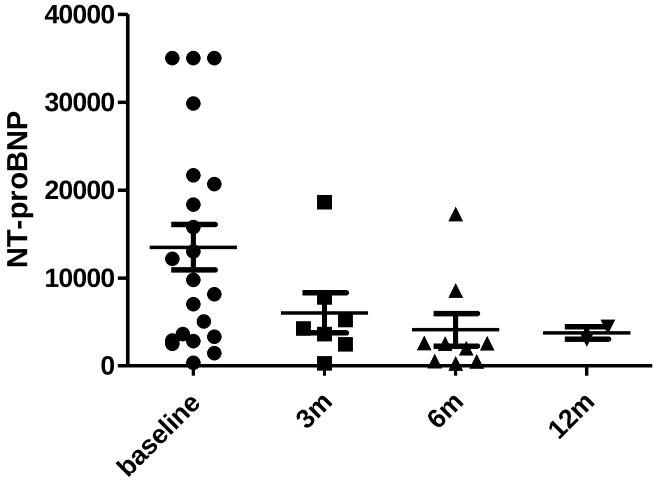

Figure S1: NT-proBNP levels of PD patients at baseline, 3months, 6months and 12 months after initiating sacubitril-valsartan.

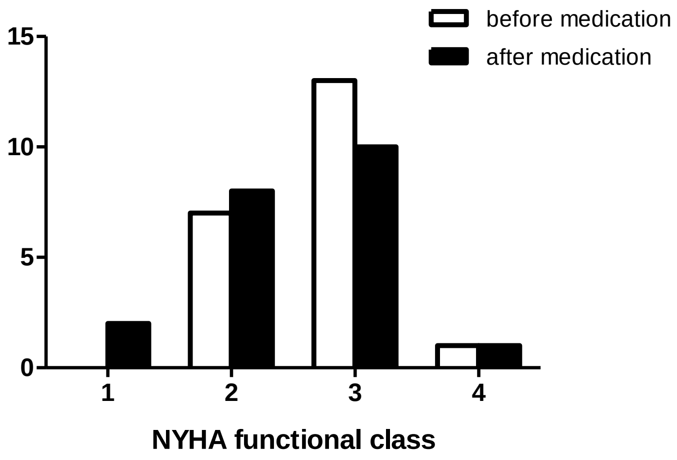

Figure S2: NYHA functional class of PD patients before and after initiating sacubitril-valsartan with observation period of 3-12 months.

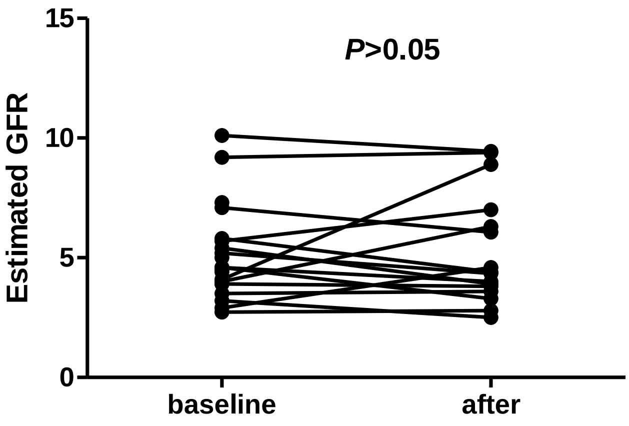

Figure S3: Estimated GFR of PD patients before and after initiating sacubitril-valsartan with observation period of 3-12 months. The Wilcoxon matched-pair signed-rank (2 samples) tests was applied to compare self-matching data of eGFR.
